# Photosynthetical activity modelisation of olive trees growing under drought conditions

**DOI:** 10.1038/s41598-019-52094-9

**Published:** 2019-10-29

**Authors:** Abderrahman Sghaier, Jari Perttunen, Risto Sievaènen, Dalenda Boujnah, Mohamed Ouessar, Rayda Ben Ayed, Kamel Naggaz

**Affiliations:** 1 Faculty of Sciences of Gabes, University of Gabes, 6072 Gabes, Tunisia; 2 Arid Regions Institute (IRA), 4119 Médenine, Tunisia; 3 Finnish Forest Research Institute, Vantaa Research Station, P.O. Box 18, 01301 Vantaa, Finland; 4 Laboratory for Productivity Improvement of the Olive Tree and Quality of Products, Institute of the Olive Tree, Specialized Unit of Sousse, Tunisia. Address: B.P. 14, 4061 Sousse, Tunisia; 5 Laboratory of Molecular and Cellular Screening Processes, Center of Biotechnology of Sfax, Tunisia. Address: B.P 1177, Sfax, 3018 Tunisia

**Keywords:** Climate and Earth system modelling, Environmental impact

## Abstract

Predicting photosynthetic production in olive trees is a key feature in managing the effect of climate change on arid areas. Functional-structural plant modelling is a promising tool for achieving this goal. We used a photosynthetic sub-model that accounted for water and temperature stress and implemented it into LIGNUM model. We then conducted an experiment to validate the model at the leaf level using olive trees (*Olea europaea*) grown under various climatic condition. Then, we simulated photosynthetic production of three static olive tree models aged 1, 2, and 3 years. Results revealed a good fit between observed and predicted photosynthesis, with coefficient of determination (R^2^) values of 0.94 and 0.93 for Chemlali and Zarrazi cultivars, respectively. These results showed that the impact of water stress on photosynthetic production was marginal.

## Introduction

The olive tree (*Olea europaea* L.) is one of the most characteristic tree species in Mediterranean agro-ecosystems, and is well adapted to severe drought^1^. However, predicting the responses of olive trees to different climate scenarios remains a key challenge for agriculture. The empirical approach cannot predict novel or non-analog responses. Thus, models based on that approach are less effective when it comes to predicting crop responses to climate change^2–4^. Furthermore, the application of empirical models in regions characterized by diverse climatic conditions is often criticized. Hence, an approach is needed that: makes future projections that include novel responses is robust to generalization across regions takes account of horticultural techniques. The process-based approach can meet the first two conditions but is insufficient to model the effects of cultivation techniques such as pruning^5–7^. The functional-structural plant model, which combines process-based models with three-dimensional plant structure, can meet all three requirements^5^. This approach allows us to simulate the canopy architecture in the environment and incorporate the effect of pruning techniques, sunlight, and carbon allocation^8–12^.

Photosynthetic production is a key feature for developing an accurate functional-structural plant model, particularly in environments with an inconsistent climate^13^. As a first step, developing a static functional-structural plant model is a conservative method of predicting photosynthesis production, without the effects of other growth-related aspects, such as carbon allocation and dynamic growth patterns. The model can also explore the performance of the functional-structural tree model in response to temperature-stressed and water-stressed environments. This is the first time this model has been applied to arid land^14^. Hence, photosynthesis predictions must be consistent and accurate across a wide range of temperatures, soil-moistures, vapour pressure deficits, and light intensities. This study aimed to implement a static olive tree model into LIGNUM. We tested the validity of a parameterized Farquhar photosynthesis sub-model coupled with the LIGNUM sky model on young olive trees grown under controlled climatic conditions. In addition, we simulated the annual photosynthetic production of three different ages of static olive tree models (aged 1, 2,and 3-years-old).

## Material and Methods

### Experimental design

**Figure 1:**
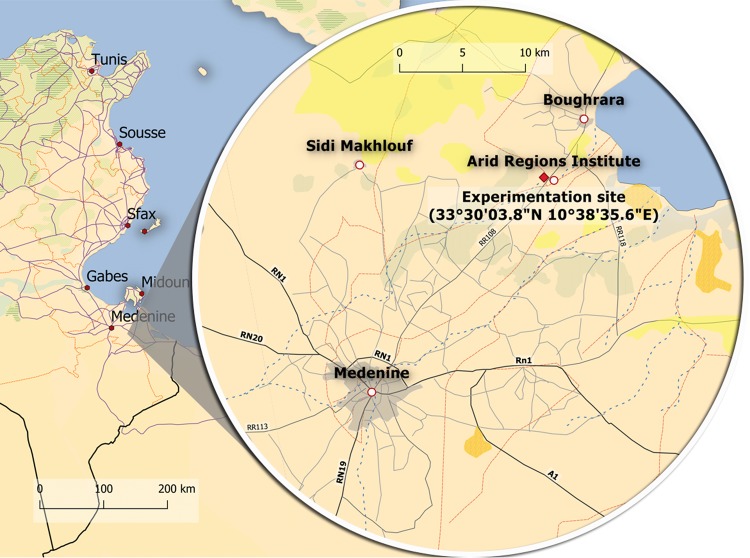
Experimental site location.

Fifty-four 1-year-old own-rooted olive tree cultivars *Olea europaea* L. cv. Chemlali and cv. Zarrazi were grown in 3-Lplastic pots containing freely drained light soil in a growth chamber at the arid region institute-Medenine-Tunisia (33°30′08.1″N 10°38′35.6″E) under three controlled climatic conditions (Condition 1: Relative Humidity (RH) = 70%, temperature (T) = 25 °C; Condition 2: RH = 70%, T = 35 °C; Condition 3: Outside: condition under uncontrolled weather conditions). The condition “outside” is defined by uncontrolled climatic conditions. In which, trees were placed outside the growth chamber. Hourly temperature and relative humidity data were provided by the Tunisian national institute of meteorology from the closest meteorological station, located in Medenine city, 18 km west of the experimentation site. The experimental site bioclimate is arid with hot and dry summers and mild winters. The experiments were conducted at a average of temperature varied from 5.6 °C to 24.7 °C, and with an average of 17 °C. Meanwhile, the average of the relative humidity was 44.7%, and fluctuated between 22% and 94%. The Fig. 1 shows the location of the experimentation site. The maps were created using QGIS software (version 3.8.1-Zanziba). Then the composed image was treated and labelled using GIMP (version 2.8.16). Three irrigation treatments were applied: the control (IRc) treatment consisted of delivering more than 100% of the available water capacity (AWC), the treatment IR1 consisted of delivering 66% of AWC and the treatment IR2 consisted of delivering 33% of AWC. The volumetric water soil content was measured twice daily in 18 pots using a time-domain reflectometer (TDR) (SM150T soil moisture probe: Delta-T Devices Ltd., Cambridge, UK). Photosynthesis was measured every 30 min using an LCI portable photosynthesis system from sunrise to sunset for all treatments according to the regime mentioned in the Table 1.

**Table 1:** Photosynthesis measurement schedule according to different leaf positions and shading.

Leaf position	Leaf under sun			Shadowed Leaf		
	Horizontal Leaf	Leaf at 45°	Leaf at −45°	Horizontal Leaf	Leaf at 45°	Leaf at −45°
Day of the month	24-01-2016	25-01-2016	26-01-2016	27-01-2016	28-01-2016	29-01-2016
	01-02-2016	02-02-2016	03-02-2016	04-02-2016	05-02-2016	08-02-2016
	09-02-2016	10-02-2016	11-02-2016	12-02-2016	13-02-2016	14-02-2016

The measurements were taken at three leaf positions: horizontal, 45° inclination angle with the normal vector of the leaf face pointing east, and −45° inclination angle with the normal vector of the leaf face pointing west. In addition, measurements were taken under sunny and shaded conditions, and a 1-m^2^ wooden board was used to create the shade.

### Light interception

#### The distribution of photon flux across the whole sky

In LIGNUM, the model sky Firmament was used to set the distribution of the light intensity across the sky. The amount of incoming radiation was divided into direct and diffused radiation, where the diffuse photon flux density originates from the midpoint of each sector, which was estimated by applying standard overcast sky radiation. The total diffused radiation was the sum of the diffused radiation generated from all the sectors. However, the direct radiation was generated from one sector according to the position of the sun. The upper hemisphere was divided into 201 sectors. The sun position, direct radiation, and diffused radiation were updated every hour.

#### Interception of photon flux by leaves

The amount of photon flux received by a leaf depends on intensity of the radiation, the leaf normal direction, and the shading caused by other leaves of the crown. As in earlier applications of LIGNUM to broadleaf trees, the shadow of the woody part was ignored and leaves were fitted into ellipses^6,15^. However, due to the natural elliptic shape of an olive tree, we assumed that the ellipses created were the actual leaves i.e., the degree of felling was 1. Therefore, a shading effect occurs when the light beam is intercepted by this ellipse. To evaluate the effect of shading on each leaf, the intensity of a light beam received by the leaf was reduced by 90% if it was intercepted by another leaf. Therefore, we added the cumulative shading effects as a coefficient for each leaf. The calculations were done separately for direct and diffused radiation. For the direct radiation, for each time step, the light beam was tracked from the centre of the leaf to the centre of the sector where the sun existed and then the cumulative shading effect was calculated. For the diffused radiation, the light beam was tracked from the centre of the leaf to the centre of each sector. However, since the trees were static, the calculation of this coefficient for each leaf was done only for the first-time step.

The radiation intercepted by the leaf from a light beam was the dot product of the leaf normal vector and the light beam vector:

1 $${\rm{DirRadInter}}={\rm{LeafNormal}}\times {\rm{DirRad}}$$

2 $${\rm{DiffRadInter}}=\mathop{\sum }\limits_{{\rm{n}}=0}^{{\rm{Nbr}}\,{\rm{of}}\,\sec }\,({\rm{LeafNormal}}.\mathrm{DiffRad})$$

where, DirRadInter is the direct radiation intercepted by the leaf DiffRadInter is the diffused radiation intercepted by the leaf DirRad is the direct radiation DiffRad is the diffused radiation; and Leaf Normal is the leaf normal.

Therefore, the total radiation intercepted by the leaf is:

3 $$PFF={\rm{DirRadInter}}\times {\rm{TaulDi}}+\mathop{\sum }\limits_{n=0}^{Nbr\,of\,sec}\,({\rm{DiffRadInter}}\times {\rm{TaulDiff}})$$

where, *PFF* is the total photon flux intercepted by the leaf TaulDir is the shading coefficient for the direct radiation; and TaulDiff is the shading coefficient for the diffused radiation.

#### Photosynthetic sub-model

Photosynthesis was modelled at the leaf level using a parameterized Farquhar model for olive trees. The model used a temperature function to improve the prediction of photosynthesis over the temperature range of 10 °C to 40 °C. Also, the photosynthesis model was coupled to a model of stomatal conductance to account for the effects of water stress on the photosynthesis productivity. The photosynthetic sub-model takes temperature, total photon flux intercepted by the leaf, water soil content, vapour pressure deficit, and reference stomatal conductance as inputs for each time step. Then, it calculates the hourly net CO_2_ assimilation of a leaf (A) after subtracting leaf respiration.

### Simulation

#### Weather data

Weather data were provided by the national institute of meteorology-Tunisia. The vapour pressure deficit was calculated according to following equations:

4 $$Vpsat=6.11\times {10}^{((7.5\times T)/(237.3+T))}$$

5 $${\rm{Vair}}={\rm{HH}}\times {\rm{Vpsat}}$$

6 $${\rm{VPD}}=\frac{({\rm{Vpsat}}-{\rm{Vair}})}{10}$$

where, Vpsat is the saturated vapour pressure Vair is the actual vapour pressure; and VPD is the vapour pressure deficit.

The HelioClim 5 database was used for solar radiation data^16^. Horizontal sensor data were used for the diffused radiation, and normal irradiance data, i.e., sensor normal is pointed towards the sun, were used for direct radiation. Since the data are given as Wh/m², a conversion factor (a) is used to convert it to μmol m^−2^ s^−1^.

7 $${\rm{Qmol}}={\rm{a}}\times \mathrm{Qw}\,$$

where, Qmol is radiation in μmol m^−2^ s^−1^; a is a constant equal to 2.34; and Qw is radiation in Wh/m².

#### Tree models

Three static olive tree models aged 1, 2, and 3 years were used in the simulations. All the models were based on real olive trees from which the following geometric characteristics were taken: length, diameter, segment girth, branching positions, reduction ratio, bending angles, leaf number and position. The total leaf-areas of the three static models were 0.067 m^2^, 1.421 m^2^ and 3.6 m^2^ for the one-year old, Two-years old and Three-years old trees, respectively.

## Results and Discussion

### Model and inputs validation

**Figure 2:**
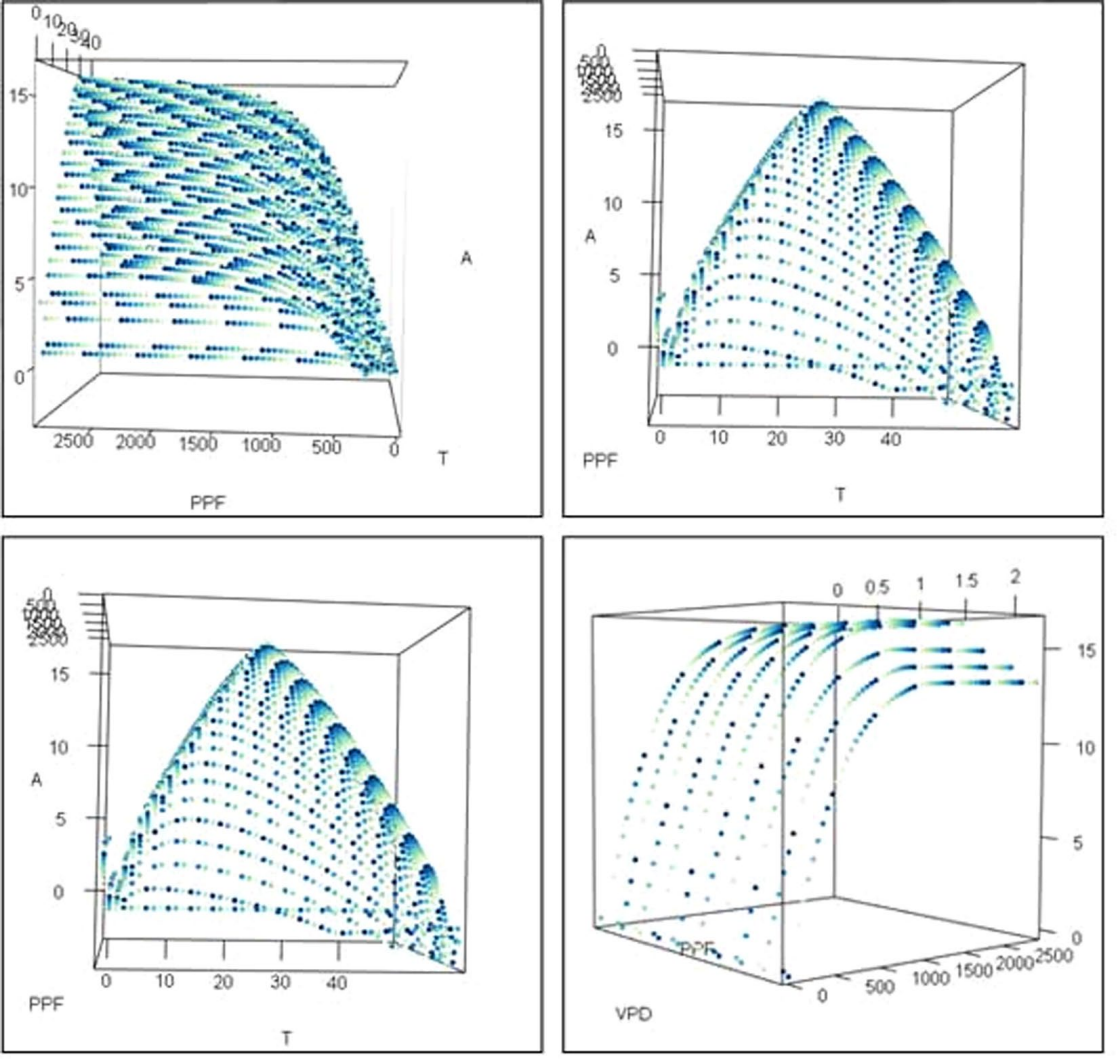
Changes in net photosynthesis production according to systematic changes in temperature, vapour pressure deficit, and photosynthetic photon flux.

A systematic simulation using all possible values of input parameters was conducted to detect possible anomalies in the calculation processes or the parameters used in the model (e.g., units used, coding glitches, or inconsistency). Figure 2 shows a three-dimensional representation of the change in net photosynthetic production depending on temperature, VPD, and photosynthesis photon flux. Figure 2 shows that photosynthetic production increased to a peak at 25 °C, then started to decline. This fits the description of the temperature dependency function for C3 plants, such as solanaceae, gramineae, and fruit-trees^17–19^. Likewise, photosynthesis responses to variation in light intensity were as expected. In fact, photosynthesis followed a logarithmic curve showing that it was very responsive to the increase in light when the intensity ranged from weak to mild. However, the sensitivity decreased as the light intensity increased, until it reached a plateau. This can be explained by the saturation of electron excitation^14^. VPD-photosynthesis dependency is controlled directly by stomatal closure; when the deficit increases, the plant reacts by closing its stomata to avoid excessive water loss. This makes CO_2_ less available in the intracellular spaces^20,21^. Therefore, photosynthesis was limited, and inversely proportional to VPD (see Fig. 2).

Under uncontrolled climatic conditions, the average of the photosynthetic production for the Chemlali cultivar was 5.42 μmol m^−2^ s^−1^, 3.81 μmol m^−2^ s^−1^ and 3.48 μmol m^−2^ s^−1^ for the irrigation treatments IRc, IR1 and IR2, respectively. Meanwhile, under the same climatic conditions the average of the photosynthetic production for the Zarrazi cultivar was 5.82 μmol m^−2^ s^−1^, 4.34 μmol m^−2^ s^−1^ and 3.99 μmol m^−2^ s^−1^ for the irrigation treatments IRc, IR1 and IR2, respectively. These values were not changed for plants cultivated under 25 °C. However, the average of the photosynthetic production was decreased significantly for the plants grown under 35 °C.

**Figure 3:**
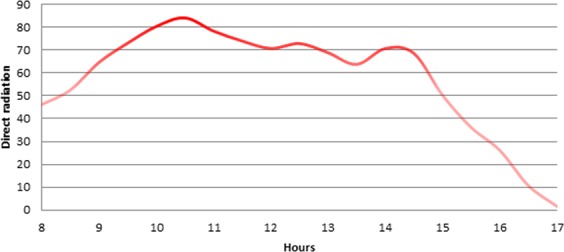
The change in mean values of direct radiation during the experiment.

Light is a sensitive environmental parameter compared to temperature or relative humidity. Light can be highly variable over a short period of time and is affected by weather conditions (e.g., cloud cover) and measurement errors (e.g., taking measurements in a shaded place). The trend in the mean values of direct radiation during the experiment is shown in Fig. 3. We observed that the general trend was normal, i.e., the light intensity increased in the morning then decreased in the evening, with a peak around midday. It is worth noting that light intensity showed mild irregularity near its maximum values from 10:00 to 15:00. This fluctuation had a small effect on photosynthesis production (as shown in the light dependency function). However under natural sunlight these minor fluctuations were normal^22^.

### Validation of the model at the leaf level

**Figure 4:**
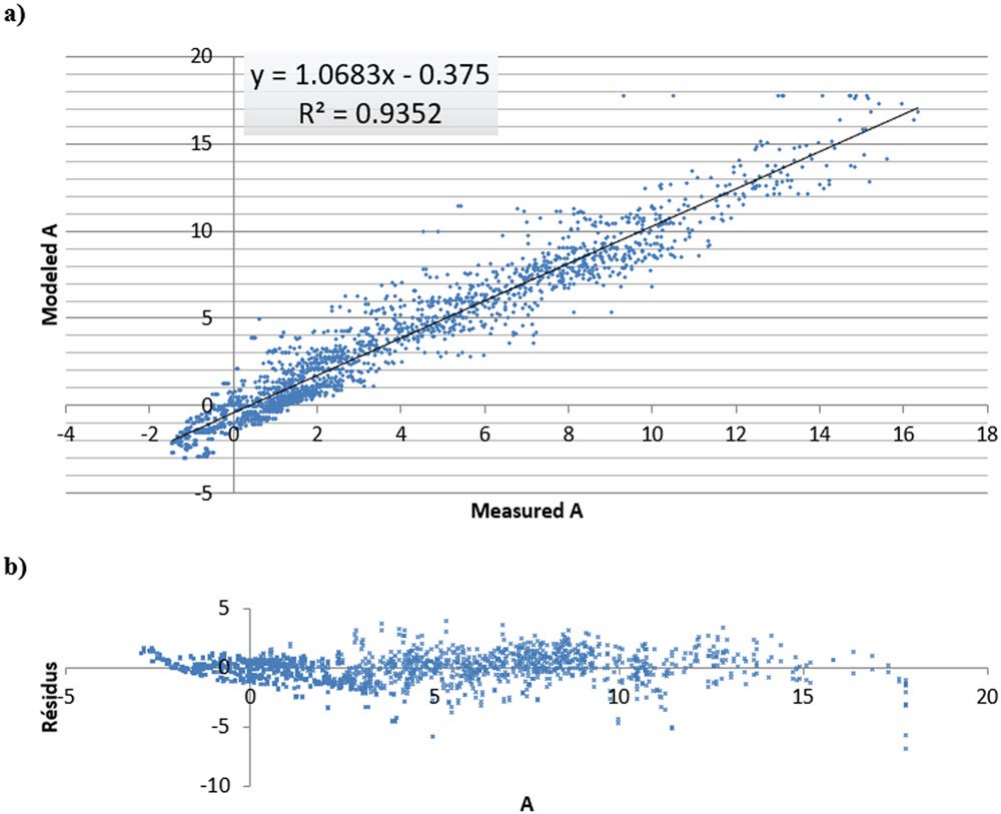
The relationship between measured and modelled net photosynthetic production. (**a**) (µmol m^−2^ s^−1^) and (**b**) residuals for all irrigation treatments and environmental conditions combined.

Figure 4a shows the comparison between measured and modelled A for both Chemlali and Zarrazi cultivars during the experiment. There was a good fit between observed and model predicted A, as indicated by the R^2^ values and Residual Mean Squared Errors (RMSE) (Table 2).

**Table 2:** Results of R^2^ and RMSE of observed and modelled leaf photosynthesis for each cultivar and irrigation dose.

	R^2^		RMSE	
	Chemlali	Zarrazi	Chemlali	Zarrazi
IRc	0.876	0.867	1.908	1.694
IR1	0.900	0.900	0.962	1.186
IR2	0.905	0.904	1.080	0.943

According to Fig. 4b (residuals of temperature), the predicted A was not biased by temperature. This indicated that the model was robust across a wide range of temperatures. Similarly, for all SWC the model explained at least 86.7% of the variation (Table 2). This clearly showed that the model predicted photosynthesis for both water-stressed and well-irrigated trees. These results confirm the findings of previous studies^23–25^.

The model accurately predicted the rate of photosynthesis under water stress. However, this is potentially misleading as the RMSE shows that photosynthesis tended to be smaller when the trees were under water stress, which can increase the R^2^ without a real increase in significance. As shown in Table 3, the regressions for individual cultivars (R^2^ Chemlali = 0.940; R^2^ Zarrazi = 0.938) were better than that for combined cultivars (R^2^ = 0.921). However, further observations revealed that the model tended to underestimate photosynthesis for the Zarrazi cultivar. Otherwise, when the photosynthetic production was close to its peak, the Chemlali cultivar recorded higher rate of photosynthesis production than the Zarrazi cultivar. This might have been due to the difference in the genetic characteristics between these two cultivars^26,27^. Thus, the Zarrazi cv appears to be a distant relative of the Chemlali cv^28^. Such relatedness would be observable some phenotypic characteristics, such as tree canopy or growth dynamic^29^. The Chemlali cv leaf area appeared to be more closely related to the Manzanilla cv, which was used to calibrate and validate the photosynthetic model. This could explain the slightly better performance of the model for the Chemlali cv^30^. These cultivars adopt different strategies to overcome water stress. Thus, the Zarrazi cv is more sensitive to water shortage, which induces a drop in transpiration rate that rapidly translates into a sharp fall in photosynthetic production^30,31^. These results showed that the cultivar determines the photosynthetic production, particularly when trees are subjected to water stress. This suggests the need for a corrective coefficient for each cultivar to accurately model this in the future.

**Table 3:** Results of the linear regression (y = a + bx), R^2^, and RMSE of observed and modelled leaf photosynthesis for each cultivar and for both cultivars combined.

Varieties	a	b	R^2^	RMSE
Chemlali	1.12	0.35	0.94	0.835
Zarrazi	1.02	0.43	0.9384	0.817
Both	0.893	0.281	0.921	1.17

**Figure 5:**
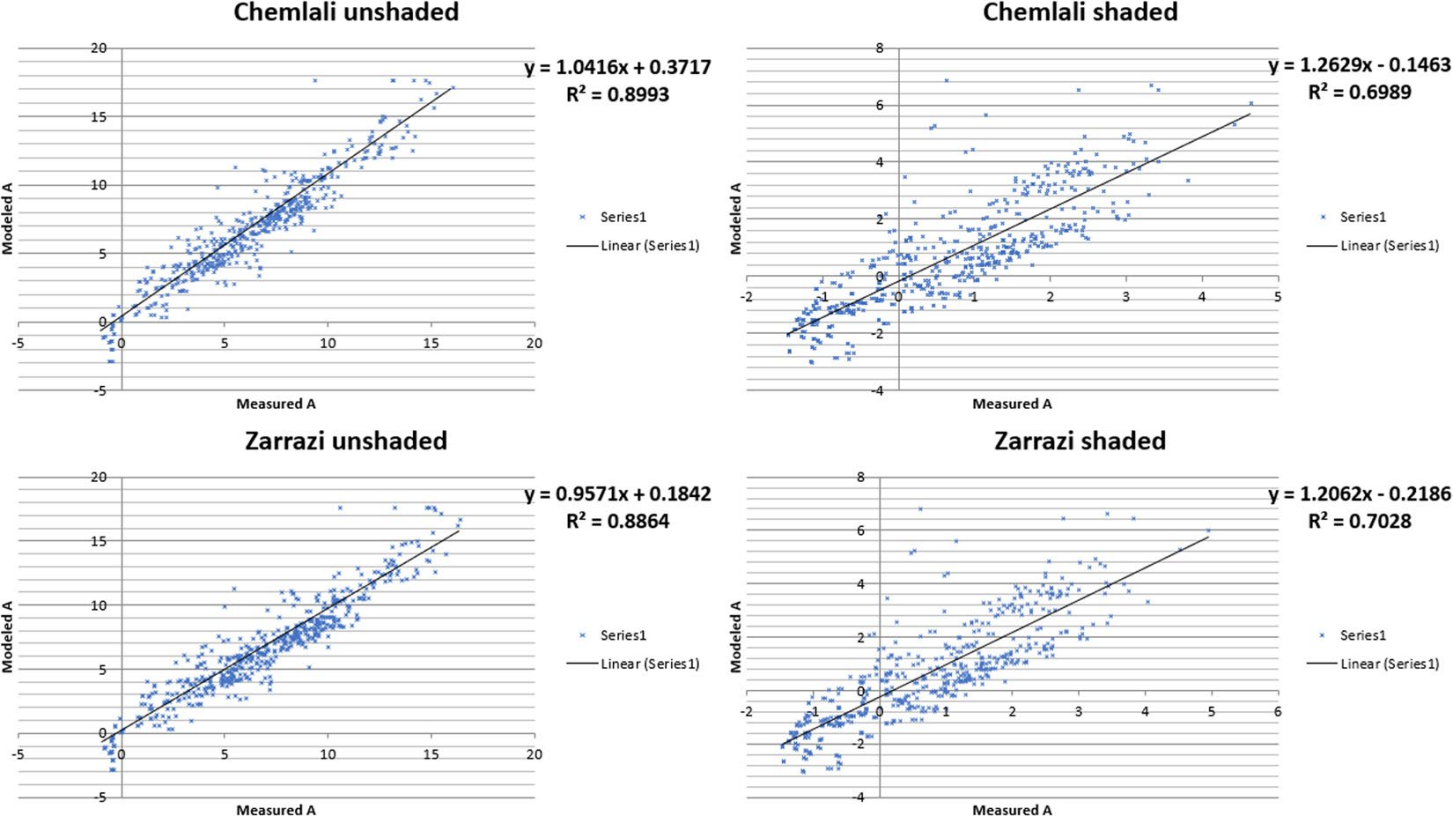
The relationship between measured and modelled net photosynthetic production (µmol m^−2^ s^−1^) for shaded and unshaded conditions for both cultivars.

Figure 5 shows that the coefficient of determination is smaller when leaves are shaded, regardless of the irrigation treatment and cultivar. This suggests a weakness in the photosynthesis sub-model when light intensity is reduced (R^2^ of Chemlali shaded = 0.6989; R^2^ of Zarrazi shaded = 0.7028). In fact, all biochemical models of leaf photosynthesis showed greater inaccuracy at low light intensity^20^. This could be explained either by the increase in Rd (rate of CO2 change in the light resulting from processes other than photorespiration) at low irradiance or the limitation of the RuBP regeneration rate at low irradiance^32,33^. Furthermore, Von Caemmerer^14^ demonstrated that the inaccuracy of photosynthesis models at low irradiance is aggravated by the thermal stress at high temperature.

**Figure 6:**
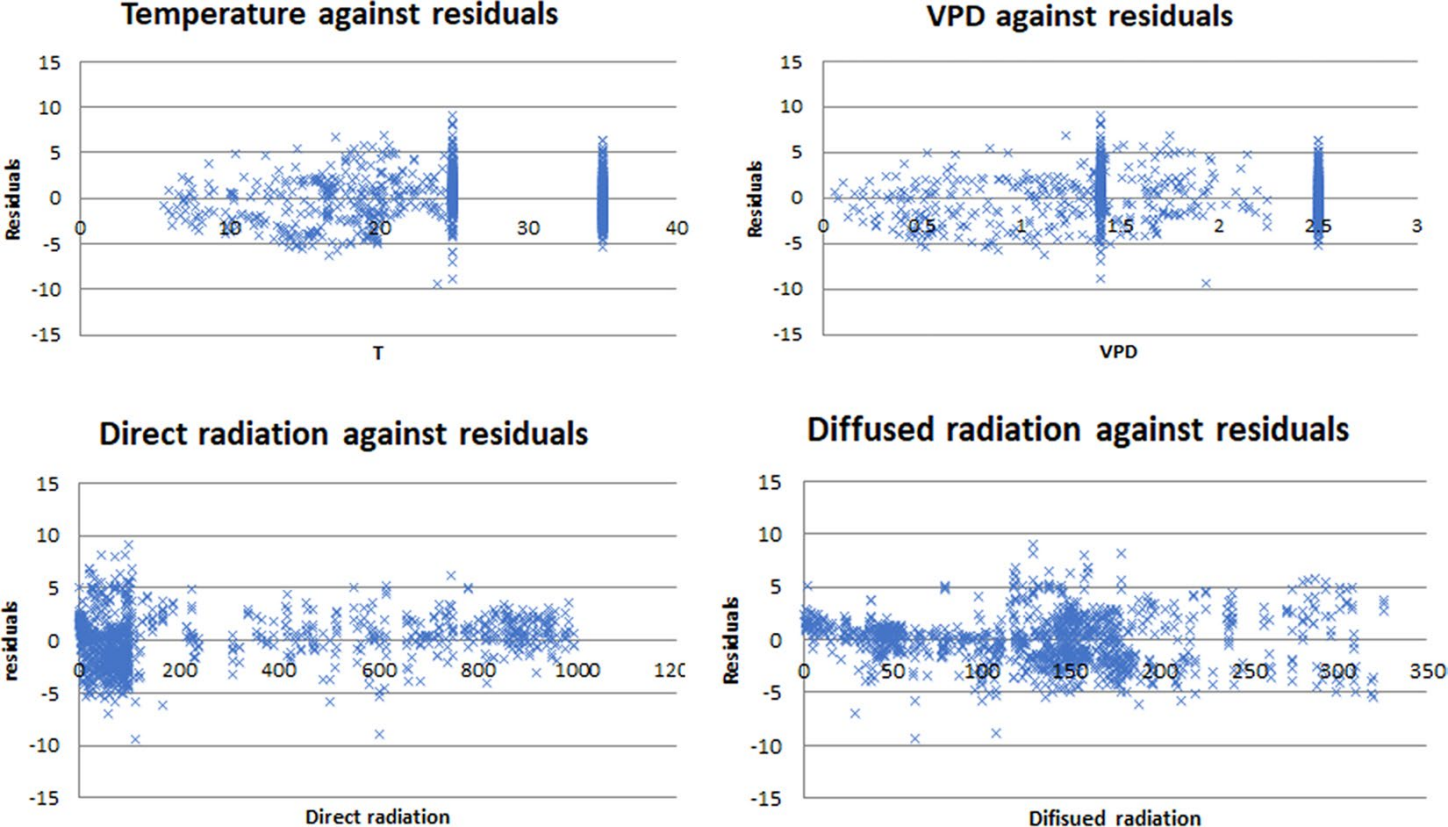
Residuals plotted against several environmental parameters.

Plotting residuals of A against direct and diffused radiation (Fig. 6) showed that only direct radiation exhibited a bias at an irradiance intensity value of ≤100. Since shading affects only direct radiation, the outcome of the shading process coincided with the biased irradiance range. Hence, we concluded that the shading calculation mechanism was less accurate, which deserves further investigation. However, at low irradiance we observed low photosynthetic production in the model.

### Simulation of carbon production using LIGNUM

The annual and seasonal net photosynthetic production model of three olive tree cultivars aged from 1 to 3 years is presented in Table 4. Simulations were run using three water soil contents (0.09, 0.14, 0.2) for the tree models. The results showed that photosynthetic production was positively correlated with the water content of the soil. For all tree ages, the photosynthetic production was reduced by 50% when the tree was subjected to water stress. The simulation showed that stressed olive trees fixed approximately the same amount of carbon regardless of the severity of the water stress. This disproportionate reaction was depicted in real olive trees under both controlled and field conditions in previous work^34^. Furthermore, this behaviour was noted for several olive cultivars^35–40^. The annual carbon produced by the trees increased exponentially with age. This could be explained by the exponential explosion in the number of leaves as the tree matures, especially in the few first years of its development. These results concur with previous findings^41–43^.

**Table 4:** Annual and seasonal net photosynthetic production of three olive tree models aged 1 to 3 years using three soil water contents.

		Winter	Spring	Summer	Autumn	Annual wet mass (Kg)
Wet Mass Kg						
1 year old	IR2	0.02	0.02	0.01	0.02	0.07
	IR1	0.02	0.02	0.01	0.02	0.07
	IRc	0.03	0.04	0.03	0.03	0.14
2-year-old	IR2	0.31	0.38	0.25	0.27	1.21
	IR1	0.32	0.40	0.26	0.29	1.26
	IRc	0.57	0.73	0.62	0.59	2.51
3-year-old	IR2	0.84	0.93	0.50	0.67	2.94
	IR1	0.86	0.97	0.53	0.70	3.06
	IRc	1.56	1.83	1.43	1.50	6.31

**Figure 7:**
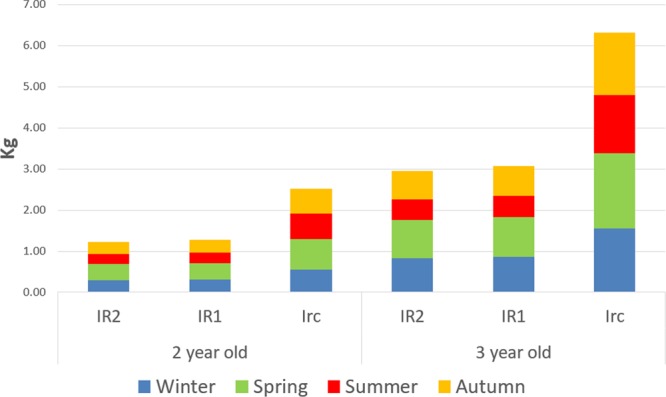
Seasonal growth of simulated trees.

Figure 7 shows that the photosynthetic productivity of trees was highest during spring and lowest during summer. This seasonality was more pronounced in water-stressed trees and less obvious in well-irrigated trees. This result was in accordance with previous work^34,39^. However, the amount of seasonality observed in vegetative growth in field conditions was higher than in modelled trees (growth had ceased in water-stressed Picholine olives). The difference between observed growth in real trees and modelled carbon production can be explained by the fact that we did not simulate carbon allocation. In summer, real trees may have indeed produced carbohydrates, but it might be allocated to storage rather than growth. Another noticeable difference between modelled and real photosynthesis was that during extreme temperatures photosynthesis needed several days (even weeks in the Picholine cv) to recover in real trees. However, the model instantly readjusted the production to the current temperature. In our study, despite the efforts to describe the impact of water stress by adopting an improved temperature function. The photosynthesis model still lacked the accuracy desired. Therefore, additional efforts are needed to improve the response of the model to long-term effects of extreme heat condition.

In the previous application of FSTM to fruit-trees little focus was given to the environmental stressors. For instance, in an FSTM model applied to mango-tree, the model was sensitive only to the amount of light received by the leaf^44,45^. Similarly, the photosynthetic production in MappleT model, an FSTM for apple-tree, was computed as a function of photosynthetic active radiation^46,47^. These models do not consider the effects of temperature, water availability or other environmental stresses that might affect photosynthesis production. Thus, their potential field application would be limited to non stress conditions. As well, in the FSTM L-peach, net photosynthesis estimation depends only on the accumulated amount of light reaching the leaf^48,49^. In this model a daily time step is adopted rather than an hourly time step. Although, L-peach consider water stress as an additional environmental factor that affect the carbon assimilation process, it was calculated at the whole canopy level using an empirical function^48^. This makes the model insensitive to the acute water and temperature stress. Therefore, it is more suitable for application that focuses on maximizing yield productivity rather than maximizing water productivity, which is the priority in arid land.

Despite this flow, the model showed high instant sensitivity to water and heat stress. Which is highly required for modelling the impact of climate change^50^. For these changes will be more pronounced in the intensity and frequency of extreme events^51,52^. VPD and temperature are crucial environmental factors that affect the stomatal behaviour, photosynthetic production and shapes the diurnal pattern of these two parameters^53,54^. These two climatic parameters are expected to be affected in the future climatic projections^55,56^. Thus, the proposed model, with an hourly time-step, is suitable to study the impact of future climatic events. The climate influences the microclimate within the canopy either directly or via feedbacks of vegetation^57,58^. With the expected climate change the well tested and mastered horticultural techniques may not be suitable in the future i.e. pruning and canopy architecture may not be suitable for the physiological need of the olive-tree^59,60^. Therefore, modelling photosynthetic at leaf level is important if the model to be applied to study the effect of climate change in fruit-tree.

### Sensitivity analysis

To perform the sensitivity analysis, we used eight parameters: Vcmax (maximum catalytic activity of Rubisco in the presence of saturating amounts of RuBP and CO_2_); GAMMA (the CO_2_ compensation point in the absence of Rd); Kc (Michaelis constants for CO_2_); Jmax (rate of electron transport for a given absorbed photon irradiance); TPU (rate of Pi release associated with trio-phosphate utilization); Rd (rate of CO_2_ change in the light resulting from processes other than photorespiration); TauL (transmission coefficient of the leaf); and Leaf area (area of the ellipse representing the leaf: SEMI_MAJOR = 0.025; SEMI_MINOR = 0.005).

The parameters (Vcmax, GAMMA, Kc, Jmax, TPU, and Rd) contributed to the simulation of physiological processes and were parameterized specifically for olive trees (Table 5). Additionally, they were very sensitive to environmental conditions. Therefore, we conducted a sensitivity analysis to investigate how photosynthesis production interacted with changes in individual parameters. TauL and Leaf area were very important for the calculation of the total photosynthesis production. Thus, the sensitivity of the model to these parameters was tested to 25% of the original values and were either added or subtracted from the studied parameters. In most cases, the output was positively correlated with variations in the parameters, except for the two parameters that had a negative effect on photosynthesis production (Rd and Gamma). However, the model appeared to be insensitive to the variation in TPU. This might be explained by the way the Farquhar model calculates photosynthesis rather than the parameter itself. In fact, TPU enables the model to mimic real world photosynthesis when it is limited solely by inorganic phosphate giving the model a physiological limit for the production of photosynthesis. To have a perfect abiotic condition for photosynthetic production is very rare, especially in the arid climates that we modelled.

**Table 5:** Sensitivity analysis of LIGNUM to variations in physiological and structural parameters of olive trees.

		tauL		leaf area		Vcmax		Jmax		GAMMA		Kc		Rd		TPU	
		+	−	+	−	+	−	+	−	+	−	+	−	+	−	+	−
3-year-old	IR2	4.39	−4.71	7.57	−13.54	0.02	−3.88	3.13	−9.24	−11.92	11.75	−1.27	0.02	−20.42	20.42	0.00	0.00
	IRc	3.41	−3.61	11.12	−15.78	0.00	−0.94	11.22	−16.49	−11.35	13.34	−0.06	0.00	−9.50	9.50	0.00	0.00
1-year-old	IR2	1.81	−2.00	19.21	−21.30	0.01	−3.69	2.83	−8.50	−10.03	9.66	−1.22	0.01	−13.60	13.60	0.00	0.00
	IRc	1.55	−1.69	19.82	−21.67	0.00	−0.93	11.12	−16.25	−10.48	12.32	−0.06	0.00	−6.77	6.77	0.00	0.00

^(+)^Change ingrowths index with 25% increase in parameter value.

^(−)^Change ingrowths index with 25% decrease in parameter value.

Combined sensitivity analyses were performed by changing two parameters at the same time (Tables 6 and 7). The parameters were selected based on their individual effects in the sensitivity analysis. The parameters selected were: Vcmax, Jmax, Rd, and Leaf area. Each one of the four parameters were tested in conjunction with another parameter, producing three possible scenarios of combined variation. Since the main objective of the study was to create a model robust under water and thermal stresses, 3-year-old trees under extreme water stress were used for the combined sensitivity analysis.

**Table 6:** Sensitivity of LIGNUM to variation in combinations of parameters using 3-year-old olive trees subjected to water treatment IR2. Each of the two parameters were combined for sensitivity analysis.

	leaf area	Vcmax	Jmax	Rd
3-years-old, IR2				
leaf area		**11**.**12**	**23**.**79**	**−0**.**77**
Vcmax	−16.56		**11**.**57**	**−9**.**50**
Jmax	−29.49	−16.49		**1**.**71**
Rd	−8.64	8.56	−6.99	

BOLD = INCREASE +25% for both parameters while *italic decrease* by −25% for both parameters.

**Table 7:** Sensitivity of LIGNUM to variation in parameter combinations using 3-year-old olive trees subjected to water treatment IR2.

	leaf area −25%	Vcmax −25%	Jmax −25%	Rd −25%
3-years-old, IR2				
leaf area +25%		10.06	−7.55	23.00
Vcmax +25%	−15.78		−16.49	9.50
Jmax +25%	−6.44	2.67		20.72
Rd +25%	−22.91	−10.44	−25.99	

Each of the two parameters were combined for sensitivity analysis. Numbers in the matrix indicate the total photosynthesis change with 25% increases in column head parameters and 25% decreases in row head parameters.

The combined analyses showed that variation in two parameters in the same direction greatly influenced photosynthetic production. For example, the increase in both the parameters Jmax and leaf area enhanced photosynthesis production by 23.6%, which was greater than the variation of any single parameter.

A positive change in Rd together with a negative change in Jmax significantly reduced photosynthesis production by around 26%. Even though it appears to have been an opposing combination of changes, it had an additive effect since the Rd parameter negatively influenced A production. Changes in two parameters in the opposite direction appeared to have a cancelling effect on photosynthesis production. This was most clear between Rd/Leaf area and Vmax/Jmax. Though, in most cases, one of the parameters dominated the other one. Therefore, the opposing change in the weaker parameter only attenuated the effect of the dominant one.

## Conclusions

Our model of olive trees integrated within the original LIGNUM model framework represents the first adaptation of the plant functional-structure model for a fruit-tree. The main novelty in this version was the incorporation of field-measured meteorological data for simulating the responses of various physiological processes to environmental stressors and integrating stomatal function that simulated the water-plant relationship. This new feature was specifically parameterized to simulate olive trees under drought conditions. Thus, it allows the first functional-structural tree model to deal with environmental stressors on fruit trees. Overall, the model was a good fit to measured photosynthesis with no evidence of bias from temperature variation. It also accurately predicted the photosynthesis production for both water-stressed and well-irrigated trees. However, the model showed several weaknesses, as it tended to underestimate the photosynthesis for the Zarrazi cultivar. Also, the accuracy of the photosynthesis sub-model simulating the A production declined significantly when light intensity was reduced. Moreover, the photosynthesis sub-model still lacked the accuracy at extreme temperatures. These flaws need to be investigated further and accounted for in the next version of the model.
